# Supplementary material for: Enhanced detection of accounting fraud using a CNN-LSTM-Attention model optimized by Sparrow search
Source: PeerJ Comput Sci. 2024 Nov 26;10:e2532. doi: 10.7717/peerj-cs.2532 (PMC11623018; doi:10.7717/peerj-cs.2532)
Supplement: Supplemental Information 2 [file peerj-cs-10-2532-s002.pdf]

Instructions for Downloading the Dataset

Please be aware that the content on the CSMAR database website is presented in Chinese.

Step 1:

Visit the CSMAR database website: https://data.csmar.com/

Create an account, then log in using your credentials.

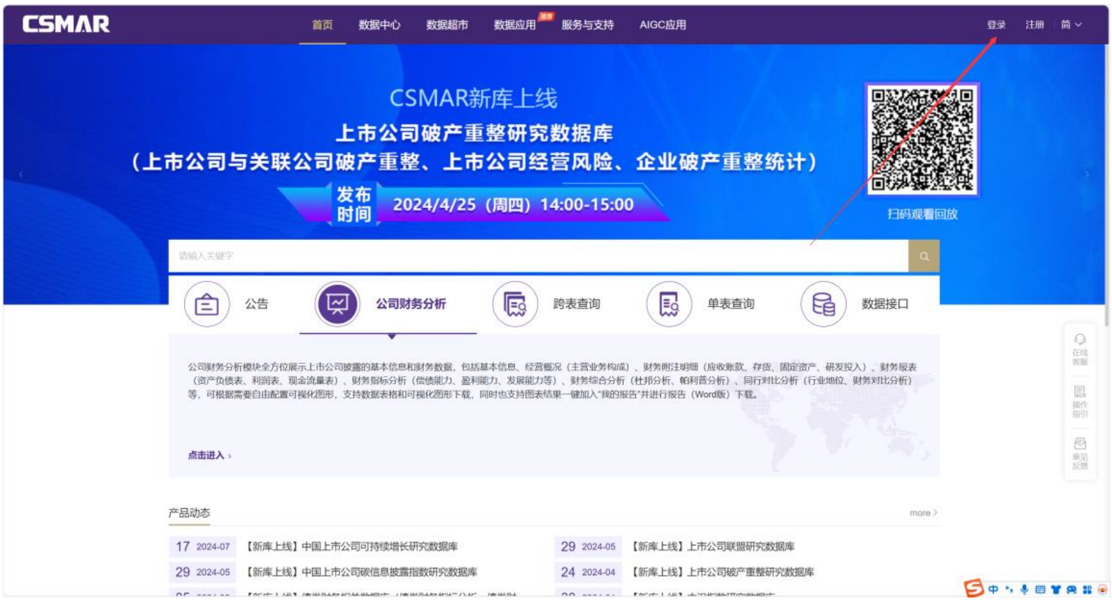

Step 2:

Once logged in, your account name will appear. Then, click on "Data Center."

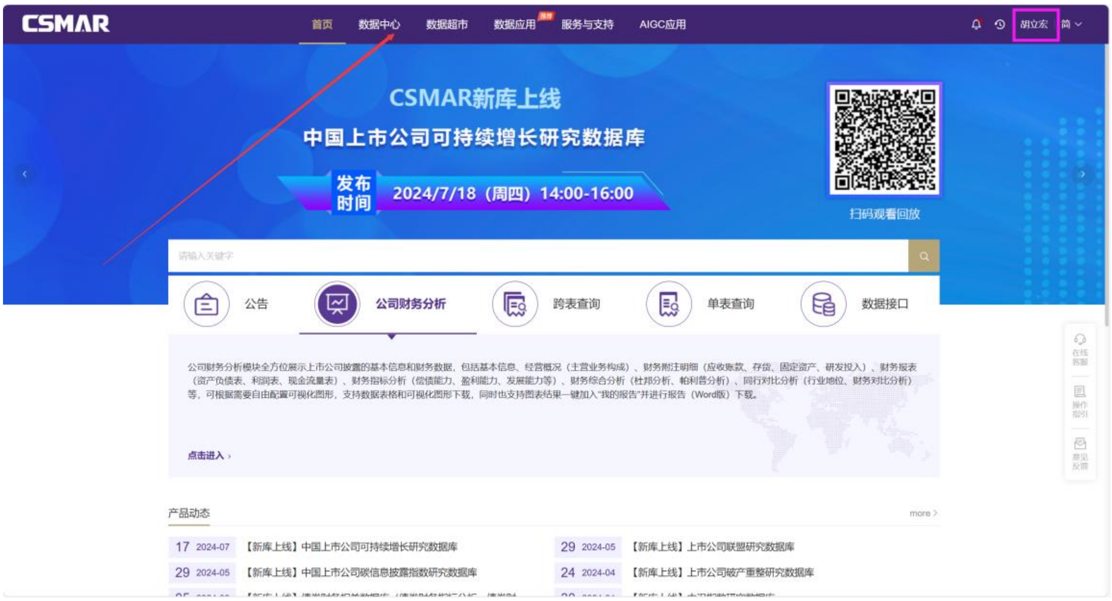

Step 3:

Filter the company data and compute statistics for the financial indicators. Select data for the desired year and include the necessary indicators.

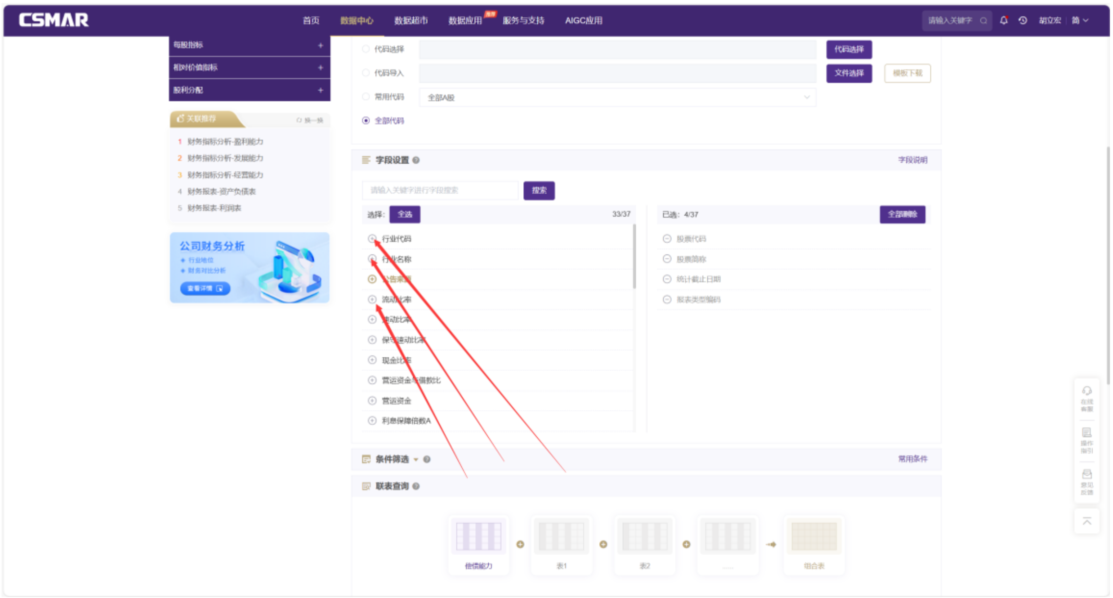

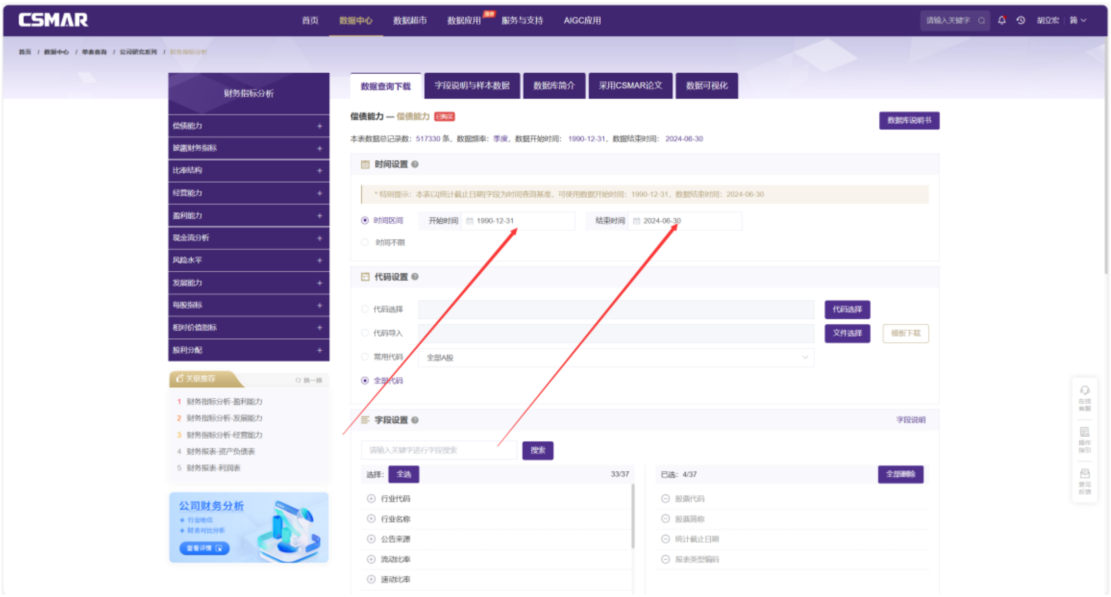

Step 4:

Download the selected data indicators.

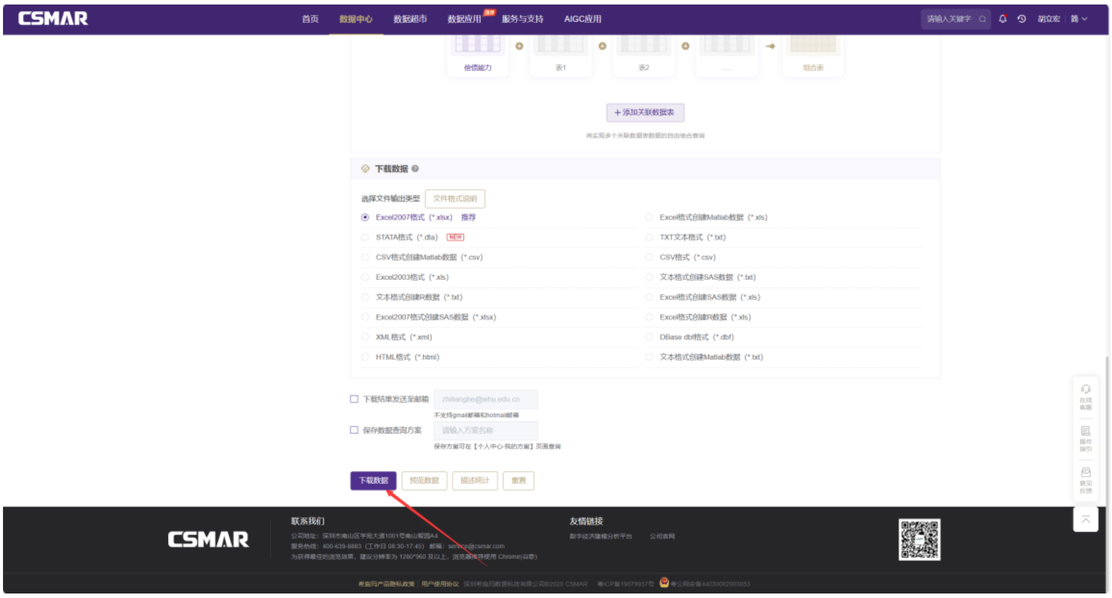

For example, using the "Operating Leverage" variable.

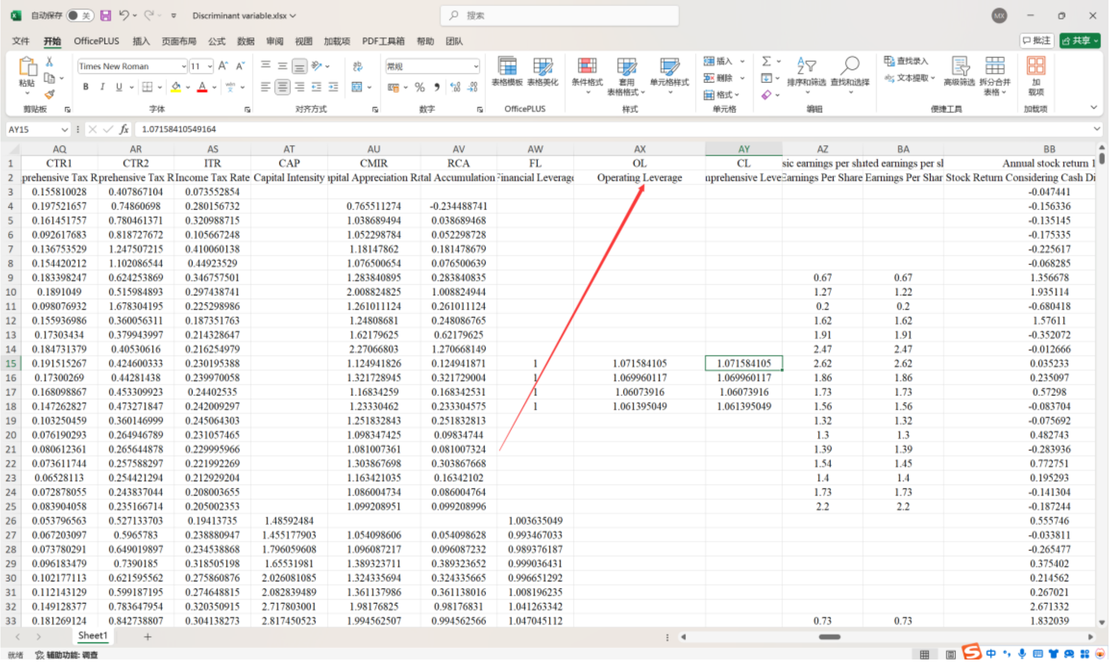

	AQ	AR	AS	AT	AU	AV	AW	AX	AY	AZ	BA	BB
1	CTR1	CTR2	ITR	CAP	CMIR	RCA	FL	OL	CL	sic earnings per sh	ited earnings per sl	Annual stock return 1
2	prehensive Tax R	prehensive Tax R	Income Tax Rate	Capital Intensity	ıpital Appreciation R	tal Accumulation	'inancial Leverage	Operating Leverage	nprehensive Leve	Earnings Per Share	Earnings Per Shar	Stock Return Considering Cash Di
3	0.155810028	0.407867104	0.073552854									-0.047441
4	0.197521657	0.74860698	0.280156732		0.765511274	-0.234488741						-0.156336
5	0.161451757	0.780461371	0.320988715		1.038689494	0.038689468						-0.135145
6	0.092617683	0.818727672	0.105667248		1.052298784	0.052298728						-0.175335
7	0.136753529	1.247507215	0.410060138		1.18147862	0.181478679						-0.225617
8	0.154420212	1.102086544	0.44923529		1.076500654	0.076500639						-0.068285
9	0.183398247	0.624253869	0.346757501		1.283840895	0.283840835				0.67	0.67	1.356678
10	0.1891049	0.515984893	0.297438741		2.008824825	1.008824944				1.27	1.22	1.935114
11	0.098076932	1.678304195	0.225298986		1.261011124	0.261011124				0.2	0.2	-0.680418
12	0.155936986	0.360056311	0.187351763		1.24808681	0.248086765				1.62	1.62	1.57611
13	0.17303434	0.379943997	0.214328647		1.62179625	0.62179625				1.91	1.91	-0.352072
14	0.184731379	0.40530616	0.216254979		2.27066803	1.270668149				2.47	2.47	-0.012666
15	0.191515267	0.424600333	0.230195388		1.124941826	0.124941871	1	1.071584105	1.071584105	2.62	2.62	0.035233
16	0.17300269	0.44281438	0.239970058		1.321728945	0.321729004	1	1.069960117	1.069960117	1.86	1.86	0.235097
17	0.168098867	0.453309923	0.24402535		1.16834259	0.168342531	1	1.06073916	1.06073916	1.73	1.73	0.57298
18	0.147262827	0.473271847	0.242009297		1.23330462	0.233304575	1	1.061395049	1.061395049	1.56	1.56	-0.083704
19	0.103250459	0.360146999	0.245064303		1.251832843	0.251832813				1.32	1.32	-0.075692
20	0.076190293	0.264946789	0.231057465		1.098347425	0.09834744				1.3	1.3	0.482743
21	0.080612361	0.265644878	0.229995966		1.081007361	0.081007324				1.39	1.39	-0.283936
22	0.073611744	0.257588297	0.221992269		1.303867698	0.303867668				1.54	1.45	0.772751
23	0.06528113	0.254421294	0.212929204		1.163421035	0.16342102				1.4	1.4	0.195293
24	0.072878055	0.243837044	0.208003655		1.086004734	0.086004764				1.73	1.73	-0.141304
25	0.083904058	0.235166714	0.205002353		1.099208951	0.099208996				2.2	2.2	-0.187244
26	0.053796563	0.527133703	0.19413735	1.48592484			1.003635049					0.555746
27	0.067203097	0.5965783	0.238880947	1.455177903	1.054098606	0.054098628	0.993467033					-0.033811
28	0.073780291	0.649019897	0.234538868	1.796059608	1.096087217	0.096087232	0.989376187					-0.265477
29	0.096183479	0.7390185	0.318505198	1.65531981	1.389323711	0.389323652	0.999036431					0.375402
30	0.102177113	0.621595562	0.275860876	2.026081085	1.324335694	0.324335665	0.996651292					0.214562
31	0.112143129	0.599187195	0.274648815	2.082839489	1.361137986	0.361138016	1.008196235					0.267021
32	0.149128377	0.783647954	0.320350915	2.717803001	1.98176825	0.98176831	1.041263342					2.671332
33	0.181269124	0.842738807	0.304138273	2.817450523	1.994562507	0.994562566	1.047045112			0.73	0.73	1.832039

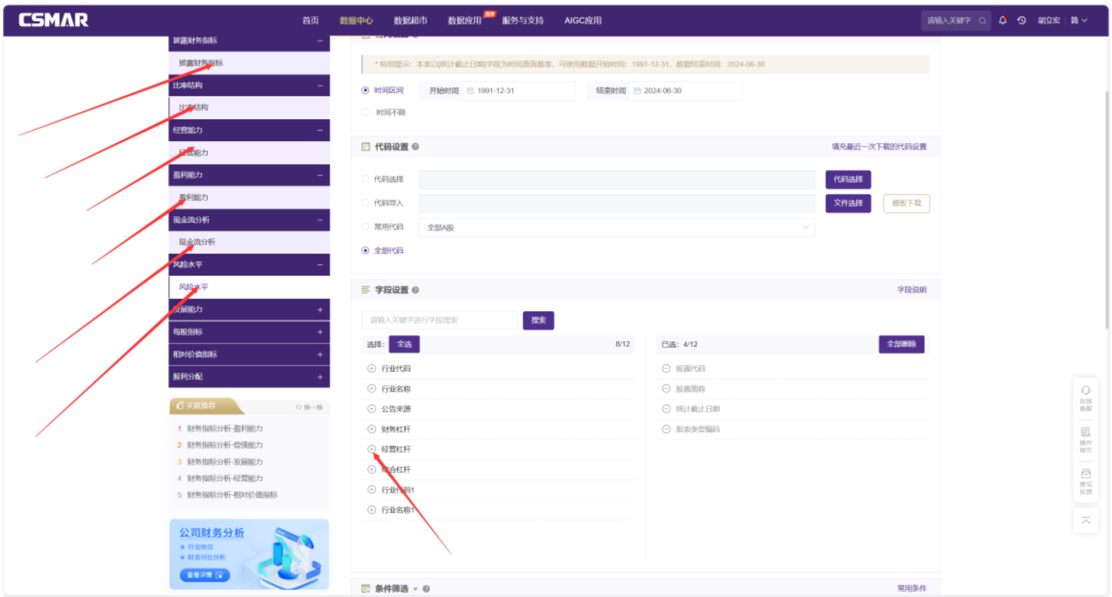

Click "Add," then export the data to an Excel file for filtering. Remove any irrelevant information.

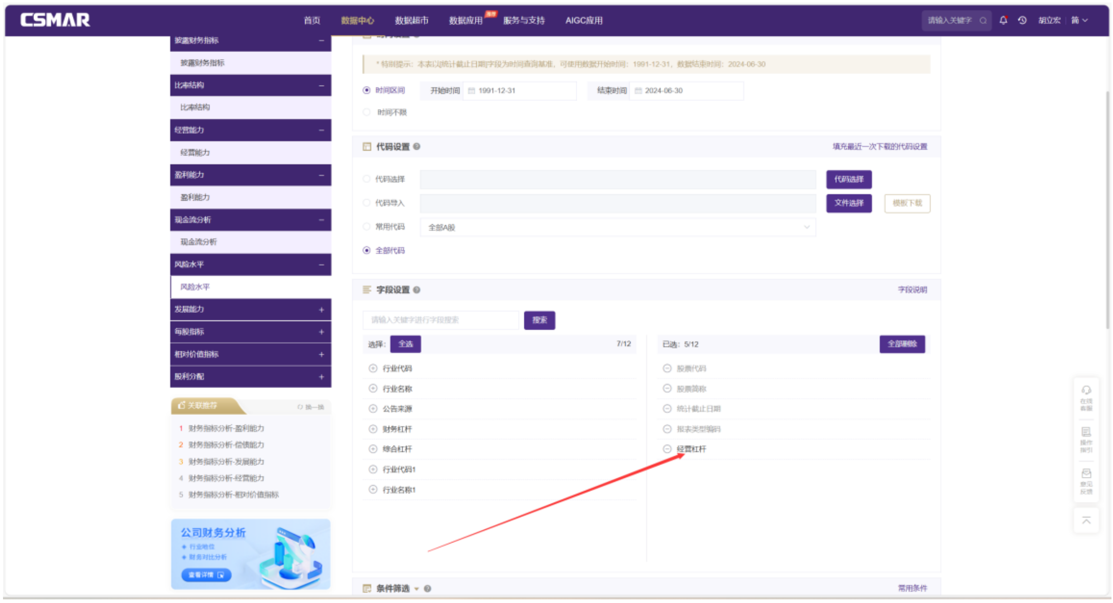

The subsequent variables follow the same repeated process. For variables with missing values, use MATLAB code to automatically remove them in batches.
